# Effect of Fe/Ni Microalloying on Interface Regulation of SiC/Al Composites: Molecular Dynamics Simulation and Experiments

**DOI:** 10.3390/ma19020283

**Published:** 2026-01-09

**Authors:** Tianpeng Song, Xiaoshuang Du, Tao Xia, Yong Liu, Jingchuan Zhu, Xuexi Zhang

**Affiliations:** School of Materials Science and Engineering, Harbin Institute of Technology, Harbin 150001, China; s2081529134@163.com (T.S.); du13298122314@163.com (X.D.); 19112354078@163.com (T.X.); zhu-jc@126.com (J.Z.); xxzhang@hit.edu.cn (X.Z.)

**Keywords:** SiC/Al composites, molecular dynamics simulation, interface regulation, mechanical properties

## Abstract

**Highlights:**

**What are the main findings?**

- Fe and Ni atoms segregate at the SiC/Al interface, blocking interfacial reactions and suppressing Al_4_C_3_ formation.
- Fe/Ni microalloying promotes dislocation proliferation and induces new dislocation types by causing lattice distortion.
- Experiments verify that Ni exhibits a superior strengthening effect, increasing the strength–plasticity product of the composite by 54%.

**What are the implications of the main findings?**

- Validates Fe and Ni as effective microalloying elements for SiC/Al composites.
- Reveals the core mechanism of microalloying element segregation in regulating dislocation behavior.
- Establishes a technical system of “molecular dynamics simulation-experimental verification”.

**Abstract:**

SiC/Al matrix composites are prone to forming brittle Al_4_C_3_ phase via interfacial reactions during fabrication, which severely limits their mechanical properties and engineering applications. Microalloying is an effective method to inhibit this brittle phase, yet the interfacial mechanism of alloying elements at the atomic scale remains unclear. Centered on molecular dynamics simulation combined with experimental verification, this study systematically investigates the laws of Fe and Ni microalloying on the interface regulation and mechanical property optimization of SiC/Al composites. Simulation results show that Fe and Ni atoms tend to segregate at the SiC/Al interface, which can suppress interfacial reactions, promote dislocation nucleation and proliferation, induce new dislocation types, and achieve the synergistic improvement of strength and ductility—with Ni exhibiting a more prominent strengthening effect. Composites prepared by the pressure infiltration-hot extrusion process show no Al_4_C_3_ phase in phase detection. Mechanical property tests confirm that Fe and Ni microalloying can effectively enhance the comprehensive performance of the materials, among which Ni increases the strength–ductility product by 54%. This study clarifies the interfacial regulation mechanism of Fe and Ni microalloying at the atomic scale, providing theoretical guidance and experimental support for the microalloying design of SiC/Al composites.

## 1. Introduction

Silicon carbide whisker-reinforced aluminum matrix (SiCw/Al) composites exhibit broad application prospects in high-end industrial fields such as aerospace and automotive manufacturing, attributed to their excellent comprehensive properties including high specific strength, high specific modulus, superior wear resistance, good thermal stability, and high fatigue strength [1,2,3,4]. The outstanding structural characteristics of SiC whiskers not only significantly enhance the thermal stability of aluminum matrix composites but also optimize their dimensional stability, further expanding the application scope of the material [5,6]. However, during the material preparation and service process, interfacial chemical reactions are prone to occur between SiC and the Al matrix, resulting in the formation of brittle Al_4_C_3_ phase. This brittle phase severely impairs the strength, elastic modulus, and corrosion resistance of the composites, becoming a key bottleneck restricting their performance optimization and engineering applications [7,8].

To address the aforementioned interfacial bottleneck, academic circles have developed various regulation strategies such as interface coating, preparation process optimization, and microalloying [9,10,11,12]. Among these, microalloying is regarded as a technical route with great engineering application potential due to its simplicity of operation, controllable cost, and ability to achieve synergistic optimization of interface characteristics and matrix properties [13,14,15,16]. Existing studies have shown that alloying elements can regulate the interface bonding state through interface adsorption, formation of interfacial compounds, or solid solution strengthening, thereby inhibiting the formation of Al_4_C_3_ [17,18,19,20]. Nevertheless, the effect of different alloying elements varies significantly; excessive addition of certain alloying elements may lead to the formation of brittle intermetallic compounds, resulting in a decrease in material strength. Moreover, the interfacial action mechanism of these elements at the atomic scale still lacks systematic elaboration [21,22,23].

To expand the selection range of alloying elements and clarify the interface regulation behavior, based on the preliminary screening of Fe and Ni elements with interface regulation potential via machine learning-accelerated first-principles calculations [24], this study further employs molecular dynamics simulation to reveal their atomic-scale action mechanism, combined with experimental verification, aiming to construct a complete technical system for the performance optimization of SiC/Al composites. The research process is as follows: Firstly, different molecular dynamics polycrystalline models are constructed and relaxed at 300 K, and the interfacial atomic distribution characteristics are analyzed by comparing the position changes in Fe/Ni atoms before and after relaxation. Secondly, the strengthening mechanism of the composites is revealed through stress–strain curve analysis and characterization of the system’s dislocation density. Finally, the composites are prepared by pressure infiltration method. Through microstructural observation and mechanical property testing, the influence laws of Fe/Ni microalloying on the microstructure and mechanical properties of SiC/Al composites are systematically investigated.

## 2. Materials and Methods

**Figure 1:**
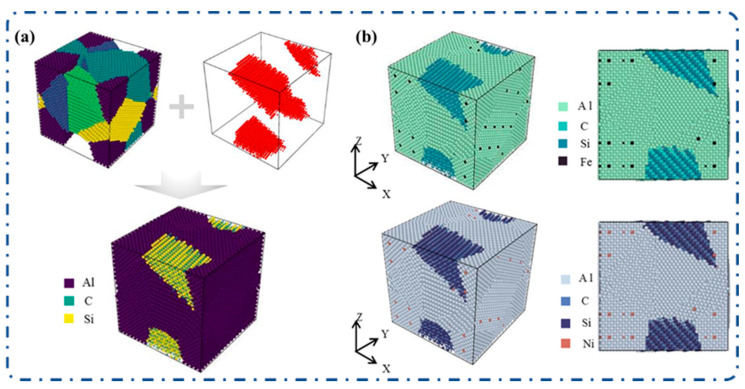
Schematic diagram of the molecular dynamics model establishment process. (**a**) Schematic of the preparation of the SiC/Al composite polycrystalline model, which is composed of a pure Al polycrystalline model and a SiC polycrystalline model; (**b**) 3D polycrystalline model of SiC/Al after Fe/Ni microalloying.

In this study, Atomsk v0.12 and Materials Studio 8.0 software were employed for model construction and data file conversion, followed by the generation of input files required for LAMMPS (Version of 2 August 2023) [25] simulations. The pure Al polycrystalline model consisted of 6 grains with different orientations, and a specific vacancy was formed by removing 1 grain. A SiC polycrystalline model with 6 grains of different orientations was constructed using the same command file, and 5 grains were removed to form a spatial structure equivalent to the aforementioned vacancy. The two models were combined to obtain a SiC/Al composite model with dimensions of 90 Å × 90 Å × 90 Å (containing a total of 42,974 atoms, including 38,930 Al atoms, 2022 Si atoms, and 2022 C atoms; Al had a face-centered cubic (FCC) structure, and SiC had a hexagonal close-packed (HCP) structure), as shown in Figure 1a. SiC/Al-Fe and SiC/Al-Ni composite models were constructed by replacing 2.5% of Al atoms with Fe and Ni atoms, respectively, as illustrated in Figure 1b. The deform command was used to perform tensile simulations on the SiC/Al molecular dynamics models. Periodic boundary conditions (ppp boundary conditions) were applied in the x, y, and z directions during the simulations, with a time step set to 0.001 ps. The potential functions were selected as follows: the embedded atom method (EAM) potential [26] was used for Al-Al interactions, the Tersoff potential [27] for Si-C interactions, and the Lennard-Jones (LJ) potential [28,29] for both Al-C and Al-Si interactions (detailed parameter settings are presented in Table S1). The models were relaxed under the isothermal–isobaric (NPT) ensemble with 20,000 relaxation steps; the tensile rate was set to 0.01/ps, and the total number of tensile steps was 50,000. The visualization of the SiC/Al composite material model and tensile simulation results was achieved using OVITO 3.12.0 software [30].

The material preparation mainly included four steps: preform fabrication, pressure infiltration, hot extrusion, and T6 heat treatment. SiC whiskers with a diameter of 5–10 μm were cleaned with distilled water and hydrofluoric acid (pH = 6), mixed and stirred with silica gel at a mass ratio of 1:1, held in a mold for 2 h, and sintered at 850 °C for 2 h to obtain a dense preform. Subsequently, 6061 aluminum alloy and Al-2Fe/Al-10Ni master alloys were melted at 800 °C, stirred at 750 °C and 1000 rpm for 0.5 h, held at 850 °C for 0.5 h, and then infiltrated into the preform under a pressure of 100 MPa for 1 min. Finally, hot extrusion was performed at a temperature of 510 °C (held for 1 h) with an extrusion ratio of 16:1. The T6 heat treatment consisted of solution treatment at 540 °C for 1 h and aging treatment at 170 °C for 6 h. Prior to characterization, samples with dimensions of 6 mm × 8 mm × 10 mm were cut by wire electrical discharge machining. The samples were then manually ground sequentially with 400#, 800#, 1500#, and 3000# metallographic sandpapers and polished with a 1.5 μm abrasive. Phase analysis of the polished samples was conducted using a room-temperature/high-temperature multi-functional X-ray diffractometer (XRD, X’PERT, Almelo, The Netherlands) with a scanning range of 10° to 90° and a step size of 5°. Field emission scanning electron microscopy (SEM, Gemini 560, Carl Zeiss AG, Oberkochen, Germany) was used to observe the sample morphology and tensile fracture surfaces, and energy-dispersive spectroscopy (EDS) was employed to analyze the types and contents of elements in microregions. The materials were ground into thin slices with a thickness of 70 μm, and a 3 mm diameter area was selected for ion thinning. The thinned samples were further analyzed for microstructure using a transmission electron microscope (TEM, Tecnai G2 F30, FEI Company, Hillsboro, OR, USA). Finally, tensile tests were performed on the samples at room temperature using an electronic universal testing machine (MTS 810 Material Testing System, MTS Systems Corporation, Eden Prairie, MN, USA) with a maximum load of 10 kN, a tensile rate of 0.5 mm/min, and a gauge section size of 18 mm × 5 mm × 1.5 mm.

## 3. Results and Discussion

### 3.1. Molecular Dynamics Simulation

**Figure 2:**
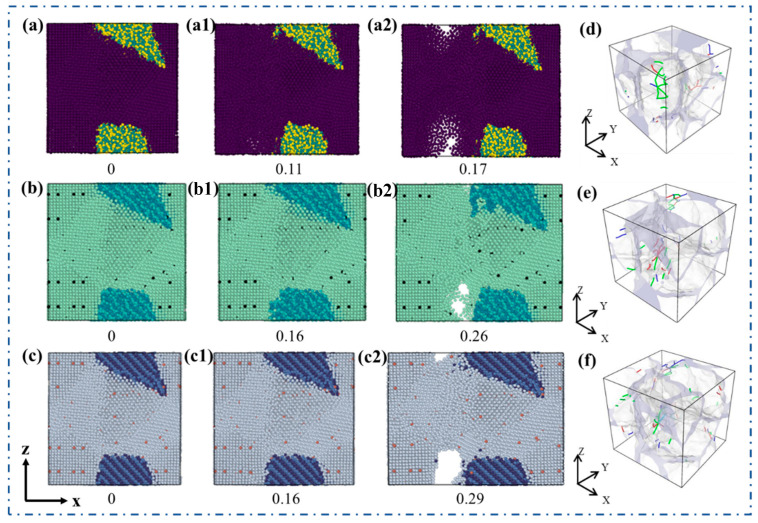
Model performance at different strain stages during tension at 300 K. (**a**–**a2**) SiC/Al composite model; (**b**–**b2**) SiC/Al-Fe composite model; (**c**–**c2**) SiC/Al-Ni composite model; (**d**) dislocation distribution at the elastic limit point of the SiC/Al composite; (**e**) dislocation distribution at the elastic limit point of the SiC/Al-Fe composite; (**f**) dislocation distribution at the elastic limit point of the SiC/Al-Ni composite.

Figure 2 systematically presents the atomic structure evolution characteristics of three composite systems (SiC/Al, SiC/Al-Fe, and SiC/Al-Ni) at different strain stages during uniaxial tension, as well as the dislocation distribution laws at the elastic limit point of each system. For the unmicroalloyed SiC/Al composite (Figure 2(a–a2)), when the strain is 0 (Figure 2a), the atoms in the model are arranged regularly, the interface between the SiC reinforcement phase and the Al matrix is tightly bonded, and there are no obvious structural defects. When the strain increases to 0.11, the elastic limit point is reached, and atoms in the matrix region begin to undergo local displacement and slight rearrangement, while the interface structure remains continuous. The dislocation distribution at the corresponding elastic limit point is shown in Figure 2d. Dislocations are mainly concentrated in the SiC/Al interface region, and the dislocation density inside the matrix is low, reflecting the characteristics of limited interface bonding strength and weak dislocation nucleation ability of the unalloyed system. When the strain is further increased to 0.17 (Figure 2(a2)), significant void defects are formed in the matrix and the material fractures.

Further analysis was conducted on the dynamic evolution of atoms during the tensile process of the microalloyed SiC/Al composites. For the SiC/Al-Fe system (Figure 2(b–b2)), it can be seen from the atomic state after relaxation (Figure 2b) that Fe atoms adjacent to SiC grains have a tendency to move towards the SiC/Al interface, while the position change in Fe atoms far from the SiC phase is small. This phenomenon confirms that Fe atoms tend to segregate near the SiC/Al interface. During the tensile process, when the strain increases to 0.16, the elastic limit point is reached, and atoms in the matrix undergo a certain degree of rearrangement. The dislocation distribution at the elastic limit point, as shown in Figure 2e, indicates that dislocations are distributed both at the interface and inside the matrix, with intertwined dislocation lines, illustrating that the introduction of the Fe element promotes dislocation nucleation and proliferation, providing support for strength improvement. Table 1 lists the dislocation density and Burgers vectors of the three composite systems at the elastic limit point. The dislocation density of the system increases to 41.96 nm^−2^, and the number of dislocations per unit volume increases to 55 dislocations/nm^3^. Compared with the unalloyed system, the dislocation density and the number of dislocations per unit volume are increased by approximately 29.3% and 37.5%, respectively. The types of Burgers vectors are still dominated by 1/6<112> partial dislocations and 1/2<110> full dislocations, but <100>-type dislocations are newly generated. This indicates that the interfacial segregation of the Fe element not only promotes the nucleation and proliferation of dislocations but also induces the formation of new dislocation types, which is related to the lattice distortion and interface stress field regulation caused by the Fe element. When the strain increases to 0.26, void defects are formed in the matrix, and the material begins to fracture, indicating that compared with the SiC/Al composite, the addition of the Fe element can delay the generation of cracks during the tensile process to a certain extent.

For the SiC/Al-Ni system (Figure 2(c–c2)), it can be observed from the atomic state after relaxation (Figure 2c) that Ni atoms adjacent to SiC grains have a more significant tendency to move towards the interface, while the position change in Ni atoms far from the SiC phase is relatively gentle, confirming that Ni atoms also have the characteristic of interfacial segregation. This behavior can further optimize the stability of the interface structure. The elastic limit point is reached when the tensile strain is 0.16, and atoms in the matrix only undergo slight local rearrangement. At this time, the dislocation density is 41.92 nm^−2^, and the number of dislocations per unit volume is further increased to 58 dislocations/nm^3^, which is approximately 5.5% higher than that of the SiC/Al-Fe system. Its Burgers vector types also include partial dislocations, full dislocations, and <100>-type dislocations, but the distribution of dislocation types is more abundant, reflecting that the Ni element has a more significant synergistic regulatory effect on the interface and matrix. The interfacial segregation of Ni atoms not only enhances the dislocation nucleation ability but also broadens the dislocation slip path by optimizing the interface bonding state, thereby forming a more complex dislocation configuration. The dislocation distribution at the elastic limit point (Figure 2f) shows the characteristic of uniform distribution of dislocations at the interface and inside the matrix with higher dislocation density. This structural feature is related to the mechanism by which the Ni element optimizes the dislocation slip path and improves the interface bonding strength, and also provides direct atomic-scale evidence for the more excellent strength–ductility matching of this system. When the strain increases to 0.29, void defects are formed in the matrix and fracture occurs, but its fracture strain is significantly higher than that of the SiC/Al and SiC/Al-Fe systems, reflecting the more prominent regulatory effect of the Ni element on matrix deformation.

**Figure 3:**
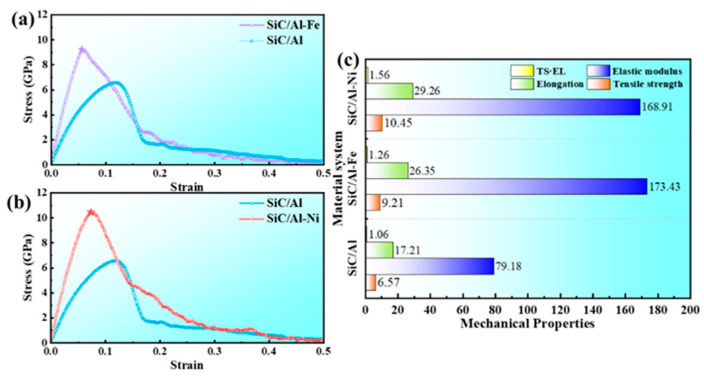
Tensile results of microalloyed SiC/Al composites. (**a**,**b**) Comparison of tensile curves of three different systems of composites; (**c**) statistical comparison of tensile performance indicators.

Figure 3a,b show the comparisons of stress–strain curves between SiC/Al-Fe, SiC/Al-Ni, and SiC/Al composites, respectively. The addition of Fe and Ni elements both significantly increases the slope of the stress–strain curve, indicating that the elastic modulus of the material is improved. In the strengthening stage, the introduction of the Fe element shifts the elastic limit point of the material to the left, but the tensile strength is significantly increased from 6.57 GPa to 9.21 GPa, with a tensile strength improvement of 40.18%, an elastic modulus improvement of 119.03%, an elongation improvement of 53.11%, and the strength–ductility product increases from 1.06 to 1.26 (Figure 3c). In the fracture stage, the stress drop rate of the SiC/Al-Fe system is significantly higher than that of the SiC/Al composite, and the stable stress value is lower than that of the SiC/Al composite. Ni microalloying has a more significant effect on improving mechanical properties, manifested as a 59.06% increase in tensile strength to 10.45 GPa, a 113.37% increase in elastic modulus to 168.91 GPa, a 70.01% increase in elongation to 29.26%, and the strength–ductility product increases to 1.56. The above results indicate that compared with the Fe element, the Ni element as a microalloying component can achieve an excellent match of significantly improved strength and ductility of SiC/Al composites.

### 3.2. Experimental Results

#### 3.2.1. Phase Composition and Microstructure

**Figure 4:**
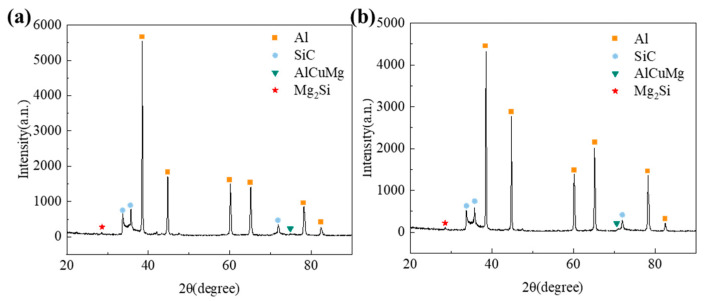
XRD analysis of as-extruded SiCw/Al-Fe/Ni composites. (**a**) XRD pattern of Fe-alloyed SiC/Al composite; (**b**) XRD pattern of Ni-alloyed SiC/Al composite.

Figure 4a,b show the X-ray diffraction (XRD) patterns of the as-extruded SiCw/Al-Fe and SiCw/Al-Ni composites, respectively. The results indicate that no characteristic diffraction peaks of the Al_4_C_3_ phase are observed in the diffraction patterns of both composites. Meanwhile, the strengthening phases AlCuMg and Mg_2_Si still exist in the matrix, demonstrating that the addition of alloying elements does not damage the original strengthening mechanism of the matrix, nor does it form new reaction phases between Fe/Ni and C/Si. This avoids the adverse effects of brittle phases on material performance.

**Figure 5:**
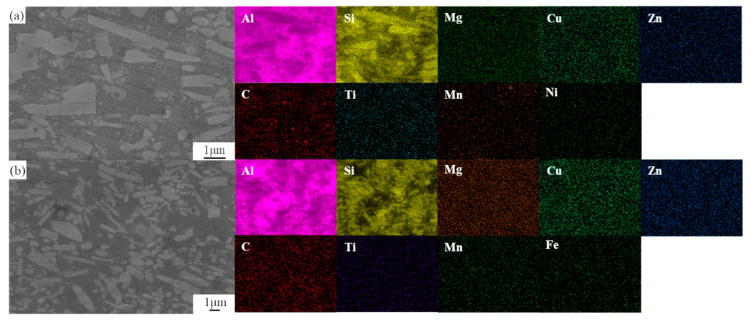
SEM images and element distributions of as-extruded microstructures. (**a**) Backscattered electron (BSE) image of SiCw/Al-Ni composite; (**b**) BSE image of SiCw/Al-Fe composite.

The scanning electron microscopy (SEM) morphologies and element distributions of the as-extruded SiC/Al-Fe/Ni composites are presented in Figure 5. It can be observed that SiC whiskers are arranged with a distinct orientation along the extrusion direction, distributed uniformly in the matrix without obvious agglomeration. Most SiC whiskers maintain intact structures, with only a small number being fractured. A small amount of fine precipitated phases exist in the matrix, which are identified as AlCuMg and Mg_2_Si phases based on the XRD results. No large precipitated phases, pores, cracks, or other defects are observed, indicating a stable material preparation process and excellent microstructure quality.

**Figure 6:**
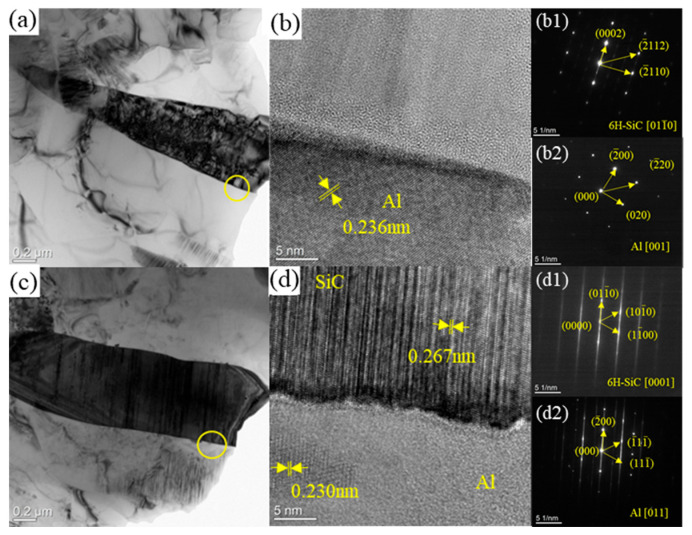
TEM images of microalloyed SiC/Al composites. (**a**) Bright-field image of SiCw/Al-Ni composite; (**b**) high-resolution TEM image of SiCw/Al-Ni composite interface; (**b1**,**b2**) selected area electron diffraction (SAED) patterns of SiCw/Al-Ni composite interface; (**c**) bright-field image of SiCw/Al-Fe composite; (**d**) high-resolution TEM image of SiCw/Al-Fe composite interface; (**d1**,**d2**) SAED patterns of SiCw/Al-Fe composite interface.

To further verify the interface structure and phase composition, high-resolution transmission electron microscopy (TEM) characterization was performed on the composites, and the results are shown in Figure 6. Figure 6a,c reveal that SiC whiskers, as the reinforcement phase, are tightly bonded to the Al matrix, with no obvious cracks, gaps, or other defects at the interface, indicating good interface bonding quality. Figure 6b is a high-resolution TEM image of the SiCw/Al-Ni composite interface: the Al matrix exhibits fine-grain characteristics, while the SiC whiskers have coarse grains. Calculations show that the interplanar spacing of SiC whiskers is 0.263 nm, and that of the Al matrix is 0.236 nm. Combined with diffraction pattern calibration (Figure 6(b1,b2)), the SiC is determined to be of the 6H-SiC type, with the zone axes of SiC and Al being [01-10] and [001], respectively. Figure 6d is a high-resolution TEM image of the SiCw/Al-Fe composite interface, showing a smooth, flat, continuous, and dense interface with good bonding. The interplanar spacing of SiC whiskers is 0.267 nm, and that of the Al matrix is 0.230 nm. Through diffraction pattern calibration (Figure 6(d1,d2)), the SiC is confirmed to be 6H-SiC, with the zone axes of SiC and Al being [0001] and [011], respectively. The TEM characterization results further confirm that no Al_4_C_3_ phase is formed at the interface of the SiC/Al composites after Fe/Ni microalloying, and the interface bonding strength is effectively improved.

#### 3.2.2. Mechanical Property Tests

**Figure 7:**
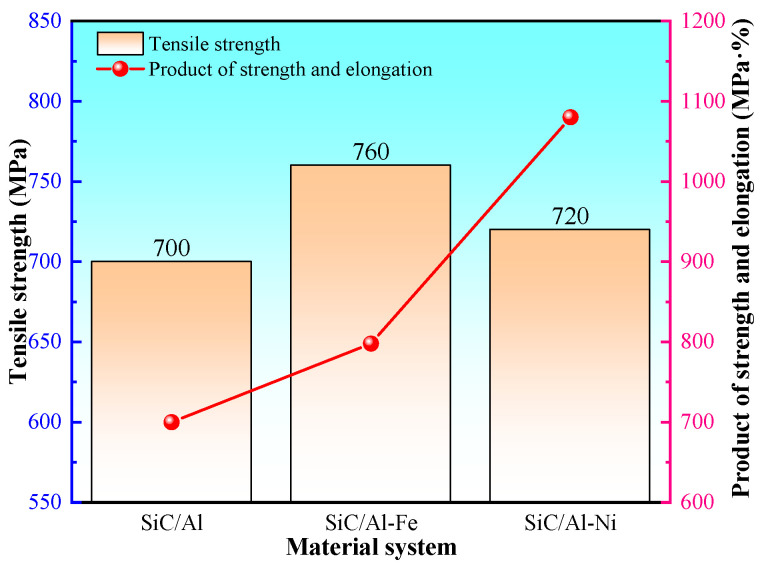
Comparison of mechanical properties of SiC/Al composites with different systems.

Tensile tests were conducted on the solution-treated and aged composites at room temperature, and the mechanical properties of the microalloyed SiC/Al composites were compared with those of the unalloyed SiC/Al composite. The results are shown in Figure 7. It is indicated that the tensile strength of the unalloyed SiC/Al composite is 700 MPa. After adding the Ni element, the tensile strength increases to approximately 720 MPa, showing a slight improvement. With the addition of the Fe element, the tensile strength reaches 761 MPa, an increase of 10% compared to the unalloyed composite. Regarding the strength–ductility product of the composites in different systems, both Fe and Ni microalloying achieve the synergistic improvement of strength and toughness of SiC/Al composites. Specifically, the addition of the Ni element increases the strength–ductility product by 54%, while the Fe element increases it by 14%. The introduction of both elements enhances the strength and toughness of SiC/Al composites to a certain extent, which is consistent with the molecular dynamics simulation results.

**Figure 8:**
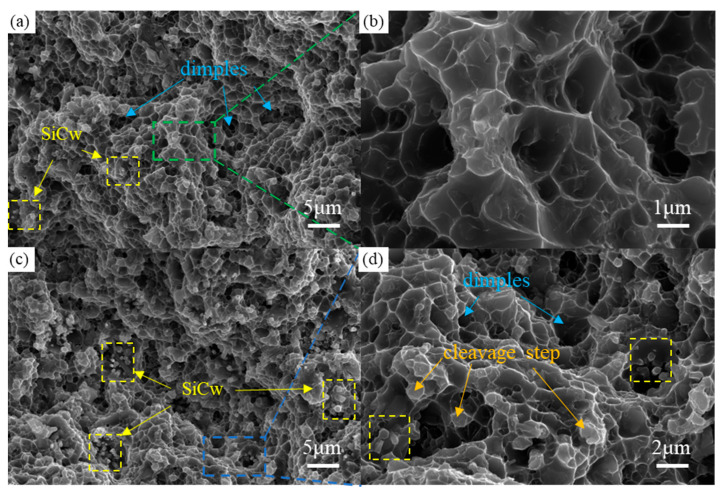
SEM images of SiC/Al composites with different systems. (**a**,**b**) Tensile fracture morphologies of SiC/Al-Ni composite. (**c**,**d**) Tensile fracture morphologies of SiC/Al-Fe composite.

Figure 8 shows the SEM images of the tensile fractures of SiC/Al-Ni and SiC/Al-Fe composites. The fracture of the SiC/Al-Ni composite exhibits a uniform mixed characteristic of dimples and tear ridges (Figure 8a) without obvious large-sized defects. High-magnification observation reveals uniformly distributed equiaxed dimples (Figure 8b), which indicates that the material has good plastic deformation capacity, confirming the positive regulatory effect of the Ni element on interface bonding and mechanical properties. The fracture of the SiC/Al-Fe composite is relatively rough with obvious undulations (Figure 8c). High-magnification images show distinct cleavage steps and dimple structures (Figure 8d), which are consistent with the characteristics of quasi-cleavage fracture. A large number of SiC whisker fracture ends are observed at the fracture (yellow areas in the figure), indicating a strong interface bonding between SiC whiskers and the matrix. The introduction of the Fe element significantly improves material strength through strengthening interface bonding and dislocation strengthening mechanisms. Based on the comprehensive fracture analysis results, the plastic deformation capacity of the SiC/Al-Ni composite is superior to that of the SiC/Al-Fe system, which is consistent with the result that the Ni element significantly improves the strength–ductility product in the mechanical property tests.

### 3.3. Comparison with Previous Works

Existing studies on the interface regulation of SiC/Al composites mainly focus on a single simulation method or experimental characterization [31,32], lacking a systematic connection between atomic-scale mechanisms and macroscopic performance verification. As the preliminary work of this study, Du et al. [24] efficiently screened alloying elements with interface regulation potential via machine learning-accelerated first-principles calculations, clarifying the advantages of these elements in terms of interfacial segregation energy and interface binding energy. However, this study only remained at the thermodynamic screening stage, failing to involve the atomic behavior evolution during dynamic tensile processes or the preparation and verification of actual materials, thus unable to reveal the interfacial action mechanisms of elements under service conditions. Xu et al. [33] adopted first-principles calculations to investigate the effects of Ti, Si, and other elements on the adhesion of Al/4H-SiC interfaces, focusing primarily on the analysis of static interfacial bonding characteristics. They failed to capture dynamic microstructural behaviors such as dislocation nucleation and multiplication during tensile deformation, nor did they verify the engineering feasibility of the calculation conclusions through experiments. In contrast to computational simulations, experiments typically require longer cycles and higher costs for certain processes. Unlike the blind trial-and-error screening mode in traditional microalloying studies, this research builds on previous machine learning screening results. It identifies core regulation pathways through simulations, reveals interfacial atomic behavior and dislocation evolution during dynamic tensile deformation, and verifies the simulated predictions of interfacial reaction inhibition and strength–plasticity product improvement through experiments. This achieves a systematic connection from atomic mechanisms to macroscopic performance, addressing the limitations of existing studies.

In terms of revealing interfacial regulation mechanisms, previous studies have mainly focused on static bonding strengthening or thermodynamic reaction inhibition. The molecular dynamics simulations in this study, for the first time, clarify at the atomic level that the regulatory effects of Fe and Ni elements not only involve the physical blocking of the reaction between Al and SiC but also induce lattice distortion and stress field reconstruction through interfacial segregation. This promotes uniform dislocation multiplication, induces the formation of new dislocation types, and significantly enriches the dislocation configuration. Compared with the multiphase interface regulation involving carbon nanotubes reported by Qiu et al. [34], this study demonstrates that microalloying with Fe and Ni can achieve the synergistic effect of dislocation multiplication and interface stabilization. Furthermore, it quantifies the differences in the regulatory effects of different elements on dislocation types through Burgers vector analysis, deepening the understanding of the dynamic mechanisms of microalloying.

Regarding the effect of performance optimization, existing studies are either limited to theoretical predictions or struggle to balance strength and plasticity. Experimental results in this study confirm that the Fe element increases the tensile strength of SiC/Al composites by 10% and the strength–plasticity product by 14%; the Ni element achieves a more significant 54% improvement in the strength–plasticity product, realizing the synergistic optimization of strength and plasticity. Dong et al. [23] reported that Ti-coated SiC tends to react at the interface to form TiC and Ti_5_Si_3_ phases. In contrast, Fe and Ni in this study inhibit the formation of Al_4_C_3_ without introducing brittle compounds, avoiding the common problem of strength–plasticity inversion. Additionally, fracture analysis reveals the dimple-tear ridge mixed characteristics corresponding to the Ni element and the quasi-cleavage fracture characteristics corresponding to the Fe element. This explains the performance differences from the perspective of fracture mechanisms, providing experimental basis for the precise selection of alloying elements.

## 4. Conclusions

In this study, molecular dynamics simulation was employed to reveal the atomic-scale mechanism of Fe/Ni microalloying on the interface regulation of SiC/Al composites, and combined with experimental verification, theoretical simulation, and experimental support were provided for the microalloying design of SiC/Al composites.

Molecular dynamics simulation clarified the intrinsic nature of interface regulation by Fe/Ni microalloying at the atomic scale: both Fe and Ni atoms exhibited a strong tendency to segregate at the SiC/Al interface, which could effectively block the interfacial reaction channel between SiC and the Al matrix and avoid the formation of brittle Al_4_C_3_ phase. Meanwhile, the interfacial segregation of alloying elements induced lattice distortion and stress field regulation, significantly promoting dislocation nucleation and proliferation, increasing dislocation density, and optimizing dislocation slip paths, thus achieving the synergistic improvement of material strength and ductility.

Simulation data showed that the Ni element had a better effect on regulating dislocation configurations and optimizing mechanical properties than the Fe element, which could increase the strength–ductility product of SiC/Al composites to 1.56. Experiments verified the reliability of the conclusions from molecular dynamics simulation. The prepared SiC/Al-Fe and SiC/Al-Ni composites were confirmed to have no Al_4_C_3_ brittle phase formation, with tight interface bonding between SiC whiskers and the Al matrix and uniform and dense microstructure. Mechanical property tests indicated that the Fe element increased the tensile strength of SiC/Al composites by 10% and the strength–ductility product by 14%, while the Ni element improved the strength–ductility product by 54% and showed better balance between strength and ductility, providing an efficient and feasible microalloying scheme for SiC/Al composites.

It should be noted that this study has certain limitations: only the regulatory effect of Fe/Ni microalloying at a single content is explored, the influence of element content gradients and the optimal range remain unclear, and the research focuses on the role of individual elements without involving the synergistic regulation mechanism of multiple elements, nor conducting systematic evaluation of key engineering properties such as fatigue and wear. For future research, simulations and experiments of Fe/Ni with multi-content gradients can be designed to clarify the content–performance structure–activity relationship, and the synergistic regulation mechanism of several or multiple elements can be explored in combination with previous machine learning screening results. Additionally, multi-dimensional performance tests under service environments should be expanded, and the coupling method of simulation and experiment optimized to improve the accuracy of the correlation between atomic-scale mechanisms and macroscopic performance, thereby providing more comprehensive support for the precise design and engineering application of high-performance SiC/Al composites.

## Figures and Tables

**Table 1:** Dislocation analysis of SiC/Al composites with different systems at the elastic limit point.

Material System	Dislocation Density (nm^−2^)	Number of Dislocations per Unit Volume (Dislocations/nm^3^)	Burgers Vectors
SiC/Al	32.46	40	1/6[211], 1/6[12-1], 1/6[-1-2-1], 1/6[1-12], 1/6[-11-2], 1/6[121], 1/6[-2-11], 1/2[101], 1/2[110], 1/2[-10-1], 1/2[-110], 1/2[0-11], 1/2[-21-1], 1/2[2-11], 1/2[1-10]
SiC/Al-Fe	41.96	55	1/6[112], 1/6[-2-11], 1/6[211], 1/6[-121], 1/6[12-1], 1/6[1-12], 1/6[-11-2], [100], 1/2[110], 1/2[101], 1/2[1-10], 1/2[10-1], 1/2[-10-1], 1/2[0-11], 1/2[12-1], 1/2[12-1]
SiC/Al-Ni	41.92	58	1/6[12-1], 1/6[211], 1/6[121], 1/6[-1-21], 1/6[-1-12], 1/6[1-21], 1/6[-2-1-1], 1/6[-11-2], 1/6[11-2], 1/2[110], 1/2[101], 1/2[01-1], 1/2[011], 1/2[-110], 1/2[0-11], 1/2[1-21], 1/2[-21-1], 1/2[-1-10]

## Data Availability

The original contributions presented in this study are included in the article and Supplementary Materials. Further inquiries can be directed to the corresponding author.

## Supplementary Materials

The following supporting information can be downloaded at https://www.mdpi.com/article/10.3390/ma19020283/s1. Table S1: Simulation Parameter Settings.
