# Supplementary material for: Effect of Fe/Ni Microalloying on Interface Regulation of SiC/Al Composites: Molecular Dynamics Simulation and Experiments
Source: Materials (Basel). 2026 Jan 9;19(2):283. doi: 10.3390/ma19020283 (PMC12842652; doi:10.3390/ma19020283)
Supplement: Supplementary file 1 [file materials-19-00283-s001.zip › materials-4061593-supplementary.pdf]

Supporting information for

Effect of Fe/Ni Microalloying on Interface Regulation of SiC/Al Composites: Molecular Dynamics Simulation and Experiments

Tianpeng Song, Xiaoshuang Du, Tao Xia, Yong Liu *, Jingchuan Zhu and Xuexi Zhang

[1] School of Materials Science and Engineering, Harbin Institute of Technology, Harbin 150001, China
* Correspondence: lyonghit@hit.edu.cn

This file includes:

- Supplementary Table S1

Table S1 Simulation Parameter Settings

Category	Parameter	Setup	Unit
Potential Function	Al-Al	EAM potential	-
	Si-C	Tersoff potential	-
	Al-C/Al-Si	LJ potential	-
kinetic parameters	Time step	0.001	ps
	Boundary conditions	Periodic boundary conditions	-
	Ensemble	NPT	-
	Relaxation	20000	steps
	Temperature	300	K
	Strain rate	0.01	/ps
	Total stretching steps	50000	steps
